# Supplementary material for: Yiai Fuzheng decoction inhibits triple-negative breast cancer by remodeling the immune microenvironment
Source: Front Immunol. 2025 Sep 30;16:1615631. doi: 10.3389/fimmu.2025.1615631 (PMC12518410; doi:10.3389/fimmu.2025.1615631)
Supplement: Supplementary file 2 [file DataSheet1.zip › Supplementary File 2/Treatment/D_vs_H/07_Pathway_Analysis/Pathway_Analysis/Sig_Pathways_Name/Arginine and proline metabolism.pdf]

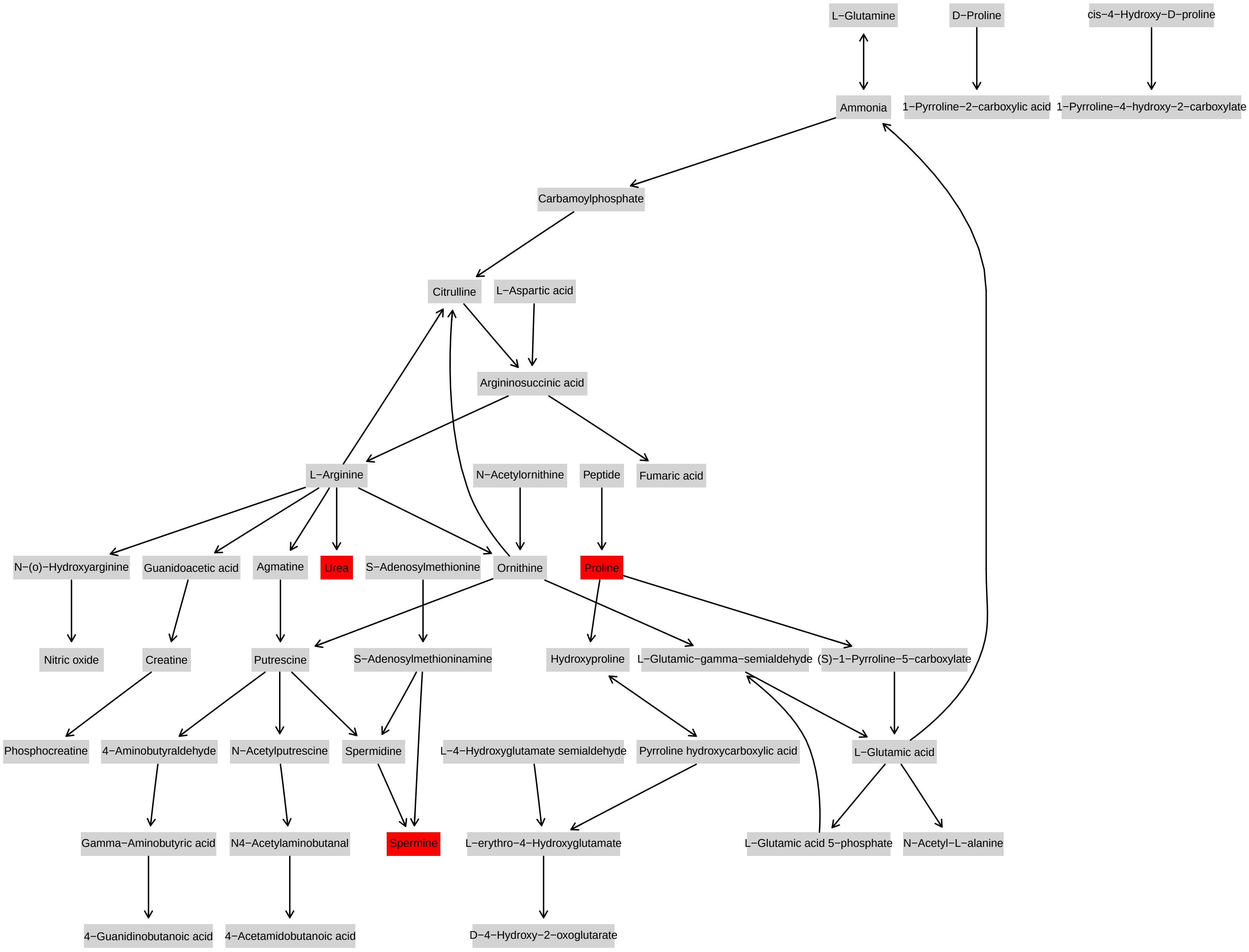

L−Glutamine
D−Proline
cis−4−Hydroxy−D−proline
Ammonia
1−Pyrroline−2−carboxylic acid
1−Pyrroline−4−hydroxy−2−carboxylate
Carbamoylphosphate
Citrulline
L−Aspartic acid
Argininosuccinic acid
L−Arginine
N−Acetylornithine
Peptide
Fumaric acid
N−(o)−Hydroxyarginine
Guanidoacetic acid
Agmatine
Urea
S−Adenosylmethionine
Ornithine
Proline
Nitric oxide
Creatine
Putrescine
S−Adenosylmethioninamine
Hydroxyproline
L−Glutamic−gamma−semialdehyde
(S)−1−Pyrroline−5−carboxylate
Phosphocreatine
4−Aminobutyraldehyde
N−Acetylputrescine
Spermidine
L−4−Hydroxyglutamate semialdehyde
Pyrroline hydroxycarboxylic acid
L−Glutamic acid
Gamma−Aminobutyric acid
N4−Acetylaminobutanal
Spermine
L−erythro−4−Hydroxyglutamate
L−Glutamic acid 5−phosphate
N−Acetyl−L−alanine
4−Guanidinobutanoic acid
4−Acetamidobutanoic acid
D−4−Hydroxy−2−oxoglutarate
